# Effects of Experimental Terrestrialization on the Skin Mucus Proteome of African Lungfish (*Protopterus dolloi*)

**DOI:** 10.3389/fimmu.2018.01259

**Published:** 2018-06-04

**Authors:** Ryan D. Heimroth, Elisa Casadei, Irene Salinas

**Affiliations:** Center for Evolutionary and Theoretical Immunology (CETI), Department of Biology, University of New Mexico, Albuquerque, NM, United States

**Keywords:** mucosal immunity, mucus, proteomics, terrestrialization, African lungfish, skin, skin-associated lymphoid tissue

## Abstract

Animal mucosal barriers constantly interact with the external environment, and this interaction is markedly different in aquatic and terrestrial environments. Transitioning from water to land was a critical step in vertebrate evolution, but the immune adaptations that mucosal barriers such as the skin underwent during that process are essentially unknown. Vertebrate animals such as the African lungfish have a bimodal life, switching from freshwater to terrestrial habitats when environmental conditions are not favorable. African lungfish skin mucus secretions contribute to the terrestrialization process by forming a cocoon that surrounds and protects the lungfish body. The goal of this study was to characterize the skin mucus immunoproteome of African lungfish, *Protopterus dolloi*, before and during the induction phase of terrestrialization as well as the immunoproteome of the gill mucus during the terrestrialization induction phase. Using LC-MS/MS, we identified a total of 974 proteins using a lungfish Illumina RNA-seq database, 1,256 proteins from previously published lungfish sequence read archive and 880 proteins using a lungfish 454 RNA-seq database for annotation in the three samples analyzed (free-swimming skin mucus, terrestrialized skin mucus, and terrestrialized gill mucus). The terrestrialized skin mucus proteome was enriched in proteins with known antimicrobial functions such as histones and S100 proteins compared to free-swimming skin mucus. In support, gene ontology analyses showed that the terrestrialized skin mucus proteome has predicted functions in processes such as viral process, defense response to Gram-negative bacterium, and tumor necrosis factor-mediated signaling. Importantly, we observed a switch in immunoglobulin heavy chain secretion upon terrestrialization, with IgW1 long form (IgW1L) and IgM1 present in free-swimming skin mucus and IgW1L, IgM1, and IgM2 in terrestrialized skin mucus. Combined, these results indicate an increase in investment in the production of unique immune molecules in *P. dolloi* skin mucus in response to terrestrialization that likely better protects lungfish against external aggressors found in land.

## Introduction

Transitioning to life on land was a fundamental step in the success and diversification of the vertebrate lineage (1). This transition imposed multiple novel challenges to vertebrates, especially at their mucosal surfaces. As a consequence, drastic physiological, histological, and molecular adaptations took place at vertebrate mucosal barriers for successful colonization of land.

The skin is the outermost organ of the vertebrate body. In aquatic vertebrates, such as teleost fish, the skin is a mucosal epithelium composed of layers of living cells coated by a mucus layer that is in direct contact with the environment. Teleost skin mucus contains many immune factors including innate and adaptive immune molecules that protect the host from invading microorganisms (2). In terrestrial vertebrates, the skin does not contain mucus-secreting cells and is cornified (3). This configuration is thought to help terrestrial vertebrates cope with desiccation stress and UV radiation.

African lungfish (*Protopterus* sp.) are a subclass of Sarcopterygian fish that are obligate air-breathers, and are the extant relative to all tetrapods (4, 5). Lungfish are evolutionarily unique organisms that have the ability to undergo conditional aestivation. Aestivation is a state of metabolic torpor, which is an adaptation for survival in areas that are subject to extreme environmental conditions (6–10). During droughts, as water evaporates from the rivers and food becomes scarce, lungfish detect environmental cues and turn them into internal signals that induce behavioral, physiological, and biochemical changes in preparation of aestivation.

There are four extant species of African lungfish: *P. aethiopicus, P. annectens, P. amphibious*, and *P. dolloi*, which implement different aestivation strategies to survive prolonged dry periods. The best-known method of aestivation is that of *P. annectens*. As water recedes, *P. annectens* will start to burrow into the mud while simultaneously secreting large quantities of mucus through its gills. This is the induction phase of aestivation. Once it has burrowed deep enough into the mud, it curls back on itself, leaving its head facing the opening of the burrow. As the water dissipates completely, the mucus/mud mixture coating around the lungfish body hardens forming a cocoon that protects the animal for months. Once encased, the lungfish ceases feeding and locomotive activities, has to prevent cell death, and sustain a slow rate of waste production until conditions become favorable (11), this is the maintenance phase of aestivation, where the lungfish can lay dormant for years. Upon the introduction of water, the lungfish instantly awakens from dormancy, leaves the mucus cocoon, and slowly swims toward the surface of the water for air (5). This is the final phase of aestivation, known as arousal, which is completed a week after water is again available and marks the return to a normal metabolic rate. *P. dolloi*, however, uses a different aestivation strategy as they do not burrow into the mud and do not appear to completely reduce their metabolic rate (12). During the induction phase, *P. dolloi* coil up on the surface of the mud while secreting mucus which, over time, turns into a dried mucus cocoon (13). Thus, *P. dolloi’s* mode of aestivation has been coined as “terrestrialization,” which is different from the complete aestivation and the full metabolic torpor observed in *P. annectens* (14, 15). Both *P. dolloi* and *P. annectens* can be terrestrialized in the laboratory setting making them ideal models to study the effects of air exposure on the vertebrate mucosal immune system (7, 9, 14).

Throughout tetrapod evolution different strategies were co-opted to maintain barrier integrity and defend against external aggressors. Antimicrobial compounds are among the most important immune molecules present in the skin of all vertebrates, both aquatic and terrestrial (16, 17). Antimicrobial proteins, lysozyme, histones, S100 proteins, and immunoglobulins (Igs) have been previously identified to be important players in the vertebrate skin immune system (18–22). Given the importance of immune molecules in skin homeostasis, tissue repair, and responses to environmental insults, we hypothesize that these molecules play a critical role during the process of terrestrialization in lungfish skin and that African lungfish will increase the amount of resources allocated to skin immunity early on in the process of terrestrialization.

The goal of this study is to characterize the African lungfish skin mucus proteome in the freshwater state, as well as the compositional change in the proteome due to terrestrialization. Our results provide a first glance to the skin proteome composition of sarcopterygian fish and its role in adaptation to terrestrial life.

## Materials and Methods

### Animals

Juvenile *P. dolloi* (slender lungfish) were obtained from Tropical Aquatics (FL, USA) and maintained in 10-gallon aquarium tanks with dechlorinated water and a sand/gravel substrate, at a temperature of 27–29°C. Fish were acclimated to laboratory conditions for a minimum of 3 weeks before being used in experiments. During this acclimation period, they were fed frozen earthworms every third day. Feeding was terminated 48 h before the start of the experiment. All animal studies were reviewed and approved by the Office of Animal Care Compliance at the University of New Mexico (protocol number 11-100744-MCC).

### Experimental Aestivation and Mucus Collection

After 3 weeks of acclimation to laboratory conditions, water in the tanks were lowered to 20 cm at 27–29°C and allowed to naturally evaporate (7). As the water level lowered, the fish entered the induction phase of aestivation and began to hyperventilate and profusely secrete mucus from their gills. This mucus combined with the substrate from the bottom of the tank encased the fish in a cocoon, which hardened after 10 days in the induction phase. In order to avoid severe dehydration due to the dry climate of New Mexico, 1–2 mL of water were sprayed on the surface of the cocoon every third day (12). Mucus was collected from the skin of one *P. dolloi* individual before the beginning of the terrestrialization experiment (named free-swimming skin mucus) and from the same individual 10 days after the start of the induction phase. At this time point, the liquid mucus actively secreted from the gills (named terrestrialized gill mucus) was aspirated with a sterile plastic Pasteur pipette. Additionally, the hardened mucus cocoon surrounding the lungfish body (named terrestrialized skin mucus) was collected by peeling it off with sterile forceps. All samples were immediately frozen at −80°C until needed for protein solubilization.

### Protein Solubilization

Mucus samples were solubilized in a protein extraction buffer (pH 7.6) made of 60 mM DTT, 2% SDS, and 40 mM Tris–HCl as previously described (23). The proteins were extracted by adding 4× the sample volume of cold (−20°C) acetone and incubating overnight at −20°C. The proteins were pelleted out and dissolved in a solution of 6 M Urea and 200 mM of ammonium bicarbonate. Protein concentrations were measured using the Pierce 660 nm protein assay (Thermo Fisher Scientific, San Jose, CA, USA).

### One-Dimensional Electrophoresis

Proteins extracted from the mucus samples were analyzed by one-dimensional electrophoresis. 12 µL of each sample was loaded into a Mini-PROTEAN TGX precast Gel at a 1:1 ratio with 2× Laemmli Sample Buffer (Bio-Rad, Hercules, CA, USA) for a final volume of 24 µL in each well. A total of 4.56 µg of protein for terrestrialized skin mucus, 3.94 µg for free-swimming skin mucus, and 1.05 µg for terrestrialized gill mucus were loaded. The proteins were separated out by the sodium dodecyl sulfate polyacrylamide gel electrophoresis (SDS-PAGE) in 1× Tris buffer, run at 120 V for 55 min, then incubated for 1 h with mild agitation in Coomassie Brilliant Blue R-250 (Bio-Rad, Hercules, CA, USA). Each lane of the gel was cut to perform proteomics analyses. Due to the presence of high-intensity band in the free-swimming skin mucus sample and a high-intensity band in the terrestrialized skin mucus sample, these two bands were first excised from their corresponding lanes and solubilized separately from the rest of the lane.

### LC-MS/MS

LC-MS/MS analysis of in-gel trypsin-digested excised protein bands or whole protein mixture-separated gel lanes (24) was carried out using an LTQ Orbitrap Velos mass spectrometer (Thermo Fisher Scientific, San Jose, CA, USA) equipped with an Advion nanomate ESI source (Advion, Ithaca, NY, USA), following ZipTip (Millipore, Billerica, MA, USA) C18 sample clean-up according to the manufacturer’s instructions. Peptides were eluted from a C18 pre-column (100 µm id × 2 cm, Thermo Fisher Scientific) onto an analytical column (75 µm id × 10 cm, C18, Thermo Fisher Scientific) using (1) a 2% hold of solvent B (acetonitrile, 0.1% formic acid) for 5 min, followed by a 2–10% gradient of solvent B over 5 min, 10–35% gradient of solvent B over 35 min, 35–50% gradient of solvent B over 20 min, 50–95% gradient of solvent B over 5 min, 95% hold of solvent B for 5 min, and finally a return to 2% in 0.1 min and another 9.9 min hold of 2% solvent B (single protein gel band analysis) or (2) a 2% hold of solvent B (acetonitrile, 0.1% formic acid) for 5 min, followed by a 2–7% gradient of solvent B over 5 min, 7–15% gradient of solvent B over 50 min, 15–35% gradient of solvent B over 60 min, 35–40% gradient of solvent B over 28 min, 40–85% gradient of solvent B over 5 min, 85% hold of solvent B for 10 min and finally a return to 2% in 1 min and another 16 min hold of 2% solvent B (whole gel lane analysis). All flow rates were 400 nL/min. Solvent A consisted of water and 0.1% formic acid. Data-dependent scanning was performed by the Xcalibur v 2.1.0 software (25) using a survey mass scan at 60,000 resolution in the Orbitrap analyzer scanning mass/charge (*m*/*z*) 400–1,600, followed by collision-induced dissociation tandem mass spectrometry (MS/MS) of the 14 most intense ions in the linear ion trap analyzer. Precursor ions were selected by the monoisotopic precursor selection setting with selection or rejection of ions held to a ±10 ppm window. Dynamic exclusion was set to place any selected m/z on an exclusion list for 45 s after a single MS/MS.

### RNA-Sequencing and Assembly

Three different transcriptomes were used to analyze the proteomic data generated in the present study. First, the pre-pyloric spleen from an experimentally infected *P. dolloi* individual was used to generate 454 pyrosequencing (Roche) transcriptome as explained elsewhere (26). Data were assembled using Roche’s GS *De Novo* Assembler. A second database was a *P. annectens* Illumina database kindly shared by Dr. Chris Amemiya and was sequenced and assembled as described in Ref. (4). The third transcriptome consisted of sequence read archive (SRA) databases from *P. annectens*, which were downloaded from the National Center for Biotechnology Information (NCBI). Sratoolkit.2.9.0 fastq-dump was used to convert the SRAs into forward and reverse paired read fastq files (27). The paired-end reads were then assembled into *de novo* transcriptomes using Trinity assembler at default parameters (28). These new transcriptomes were concatenated together into one large transcriptome, and any redundant sequences were removed using cd-hit-est at a 99% confidence level (29). The resulting transcriptome was translated into a protein database using Transdecoder-5.0.0 (28).

### Protein Identification

The protein and peptide identification results were visualized with Scaffold v 3.6.1 (Proteome Software Inc., Portland, OR, USA), a program that relies on various search engine results (i.e., Sequest, X!Tandem, and MASCOT) and which uses Bayesian statistics to reliably identify more spectra (30). Proteins were accepted that passed a minimum of two peptides identified at 0.1% peptide FDR and 90–99.9% protein confidence by the Protein Profit algorithm, within Scaffold. Tandem mass spectra were searched against the three *Protopterus* sp. transcriptomes obtained as described above. A translated protein database to which common contaminant proteins (e.g., human keratins obtained at ftp://ftp.thegpm.org/fasta/cRAP) were appended to each database. All MS/MS spectra were searched using Thermo Proteome Discoverer 1.3 (Thermo Fisher Scientific, San Jose, CA, USA) considering fully Lys C peptides with up to two missed cleavage sites. Variable modifications considered during the search included methionine oxidation (15.995 Da) and cysteine carbamidomethylation (57.021 Da). Proteins were identified at 99% confidence interval with XCorr score cutoffs as determined by a reversed database search (31).

### Proteomic Data Analysis

Transcriptome IDs for each sample were taken from Proteome Discoverer. The proteins retrieved from the excised lanes were first merged with their corresponding whole band resulting in one protein list for free-swimming skin mucus, one for terrestrialized gill mucus, and one for terrestrialized skin mucus. Tblastn searches were performed against each of the three transcriptomic data sets and resulting nucleotide sequences were then used as queries for blastx searches in NCBI (32). Human orthologous genes were assigned using AmiGO2 (33, 34), and gene ontology (GO) analysis was performed using DAVID bioinformatics database (35, 36). GO analysis of the proteome was used to identify the percentage of immune-related proteins in each sample. Venn diagrams were created in R identifying unique and common protein composition between samples (37). In order to identify proteins with known antimicrobial function, we analyzed our protein results using the list of mammalian skin antimicrobial compounds provided in the review paper by Schauber and Gallo (38).

### Histology

For light microscopy, skin samples from free-swimming and terrestrialized fish were fixed in 4% paraformaldehyde overnight, transferred to 70% ethanol, and embedded in paraffin. Samples were sectioned at a thickness of 5 µm, dewaxed in xylene, and stained using hematoxylin and eosin for general morphological analysis. Images were acquired and analyzed with a Nikon Eclipse Ti-S inverted microscope and NIS-Elements Advanced Research Software (Version 4.20.02).

### Quantitative Real-Time PCR

Skin tissue from free-swimming *P. dolloi* and terrestrialized *P. dolloi* (*N* = 3) was collected using sterile dissecting tools and placed in 1 mL of Trizol (Ambion, Life Technologies, Carlsbad, CA, USA). Total RNA was extracted from each sample, and 1 µg of RNA was synthesized into cDNA as described in Ref. (26). The resulting cDNA was stored at −20°C. The expression levels of IgM1, IgM2, IgW1, H2A, S100-A11, and neutrophil elastase (ELANE) were measured by quantitative real-time PCR (RT-qPCR) using the specific primers shown in Table 1. Phosphoglycerate kinase 1 PGK1F was used as the house-keeping gene. The RT-qPCR and statistical analysis were performed as described in Ref. (26). Data were expressed as mean ± standard error, and qPCR results were analyzed by unpaired *t*-test (*p* < 0.05).

**Table 1:** Primers used in this study.

Gene	Primer name	Primer sequence (5′–3′)	Application
PGK-1	PGK-1 F	GCAAAACAGATTGTATGGAATGGA	qPCR
	PGK-1 R	GGCACAGCATGTGGCTGTATC	
H2A	H2A F0	CTCAGAAAGGGCAATTACGCT	qPCR
	H2A R0	TGACTGGATATTGGGAAGCACT	
S100-A11	S100-A11 F0	ACGAGGAAGCTAGTAAAGATGGA	qPCR
	S100-A11 R1	ACATTTCACCAAACTCCACCT	
Neutrophil elastase	NElastase F0	AAGCCTGGAACACGTTGTCA	qPCR
	NElastase R0	CACCATTGCACACCAAAGGG	
IgW1	IgW1 F	CCTGAAGTGTACAGCAATGGTCG	qPCR
	IgW1 R0	GTTGAAGTGCCTCTAAACCATGCC	
IgM1	IgM1 F0	GCAAACCACTTGTTCCAGGGAG	qPCR
	IgM1 R0	CATGTAAATTCTTCCTCAGAG	
IgM2	IgM2 F0	GCTTAGCAACAGACTACCTTCCAGG	qPCR
	IgM2 R0	CCTGTGTACGTGCCTTTGTTAC	

## Results

### Histological Changes in the Lungfish Skin in Response to Terrestrialization

**Figure 1:**
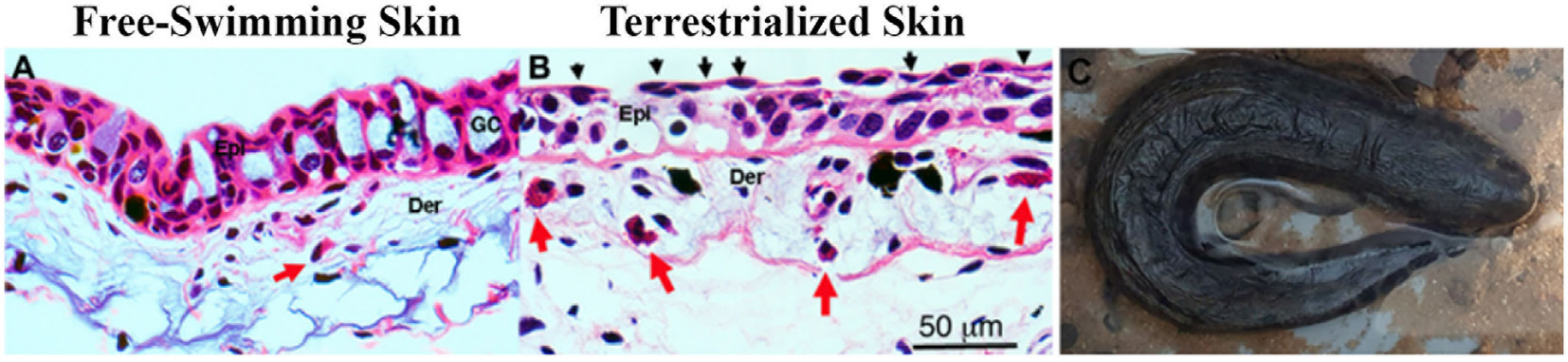
**(A)** Hematoxylin and eosin staining of free-swimming and **(B)** terrestrialized *Protopterus dolloi* skin paraffin sections. Skin sections show significant infiltration of granulocytes (red arrows) in the dermis (Der); epidermis (Epi), and goblet cells (GO). The black arrows indicate flattened keratinocytes after terrestrialization. **(C)** Image of a *P. dolloi* 4 days after the initiation of the induction phase actively secreting mucus from its gills.

Histological examination of freshwater and terrestrialized *P. dolloi* skin revealed that mucus-secreting goblet cells become exhausted from the process of terrestrialization. The epidermis becomes more compact with flattened keratinocytes on the surface creating a new barrier. We observed eosinophilic granulocytes in the dermis of both samples, but they were more abundant in the terrestrialized skin (Figure 1). These results are in agreement with previously reported changes in African lungfish skin in response to aestivation (7).

### SDS-PAGE Analysis of Lungfish Mucus Proteins

**Figure 2:**
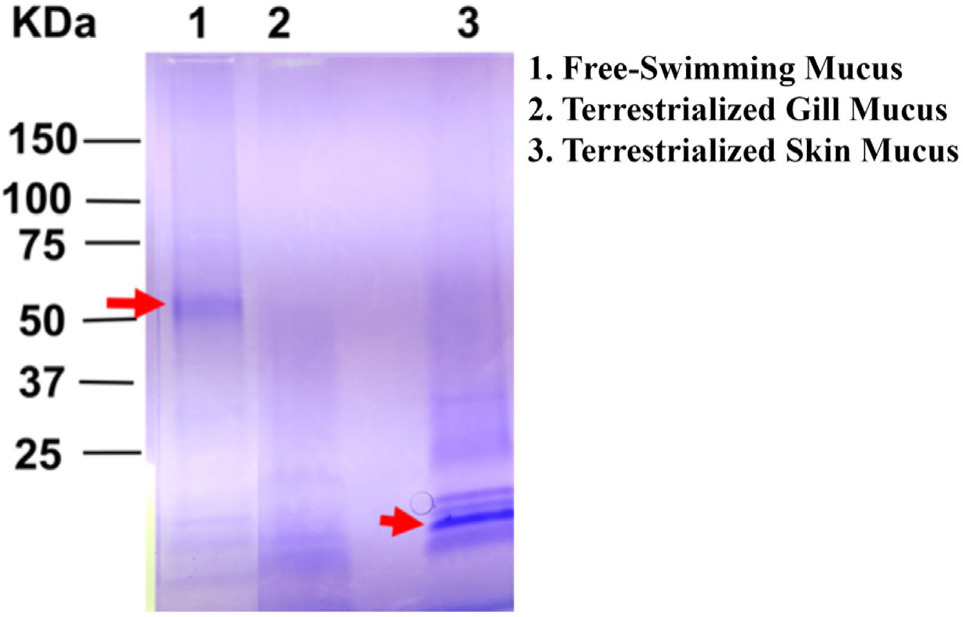
Sodium dodecyl sulfate polyacrylamide gel electrophoresis showing unique protein compositions between free-swimming (lane 1), terrestrialized gill mucus (lane 2), and terrestrialized skin mucus samples (lane 3) used for LC/MS-MS proteomic analysis. Red arrows point to high-intensity bands that were excised and analyzed by LC/MS-MS.

The overall protein composition of the three mucus samples was visualized by SDS-PAGE. The protein band patterns of each sample were unique. There was a distinct band of high intensity at ~52 kDa in the free-swimming skin mucus, while there was an intense band ~15 kDa in the terrestrialized skin mucus sample not found in the other two samples. The gill mucus sample did not contain any predominant band (Figure 2). The two bands with high intensity (arrows) were excised and analyzed separately from the rest of the lane.

### LC/MS-MS Proteomic Analysis, GO, and KEGG Pathway Analyses

**Figure 3:**
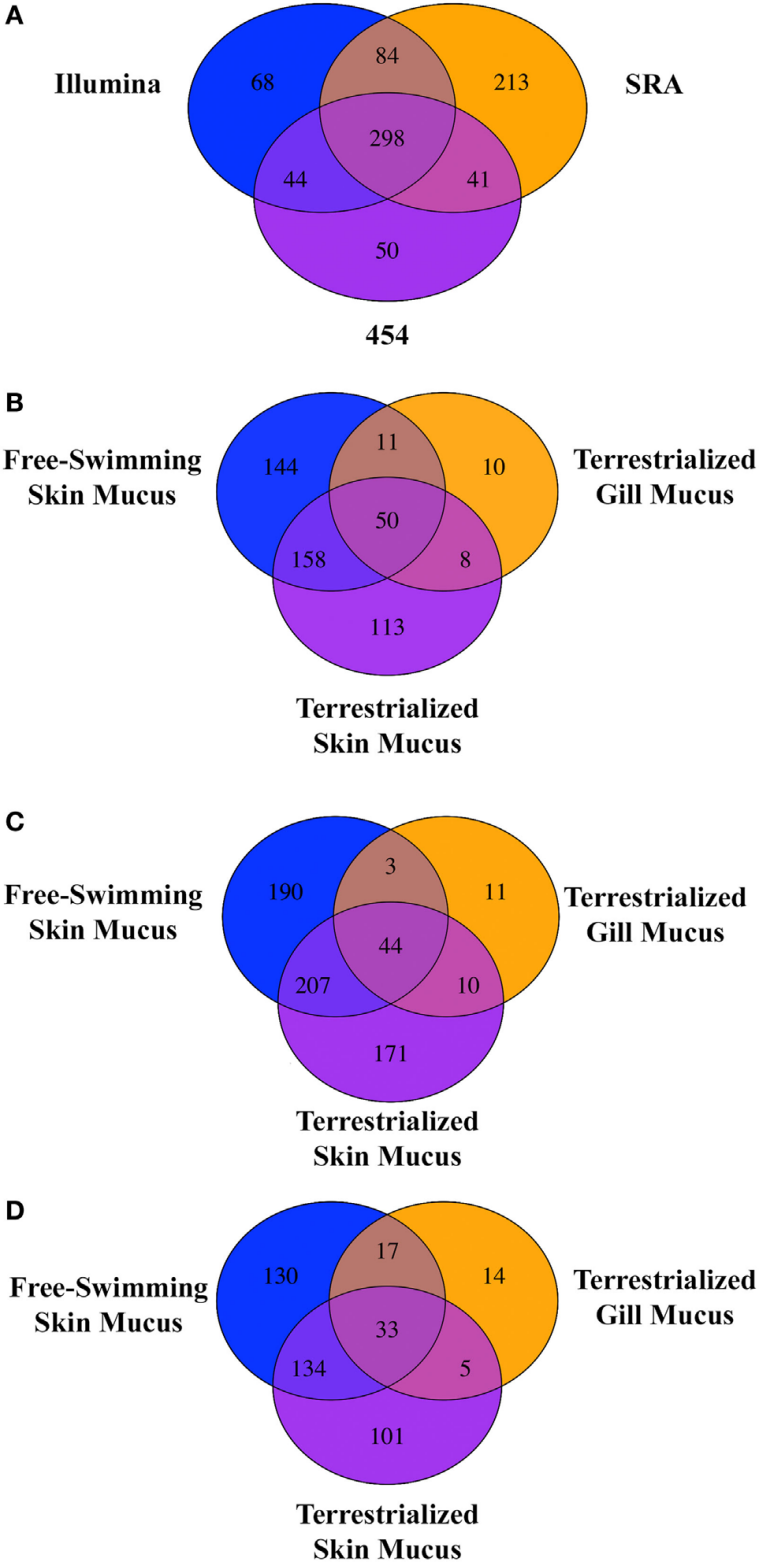
**(A)** Venn diagram showing the unique and shared proteins between the Illumina database, SRA database, and the 454 pyrosequencing database present in all three *Protopterus dolloi* mucus samples. Comparison of shared and unique proteins present in each mucus sample using the **(B)** Illumina database, **(C)** SRA database, and **(D)** 454 database.

Free-swimming, terrestrialized gill mucus, and terrestrialized skin mucus proteins from LC/MS-MS were analyzed against three RNA-seq transcriptomes, two sequenced on an Illumina platform and one sequenced using a 454 pyrosequencing platform. These translated transcriptomes resulted in a total of 53,184 protein sequences in the translated Illumina database, 269,746 protein sequences in the SRA database, and 41,351 protein sequences in the translated 454 database. The analysis returned a total of 974 proteins from the Illumina, 1,256 proteins from the SRA, and 880 proteins from the 454 database. Unique proteins were then identified using human orthologs and multiple copies of proteins were consolidated into single occurrences. As a result, 494 unique proteins were found in Illumina database, 636 unique proteins were in the SRA database, and 434 from in the 454 database. Comparing the outputted proteins from each database revealed that 298 proteins were shared when using all three databases, 213 proteins were unique proteins to the SRA database, 68 were unique to the Illumina database, and 50 were unique to the 454 database (Figure 3A). When analyzing the protein composition of each of the three mucus samples using the Illumina translated transcriptome we found 50 shared proteins among all three samples, 144 unique proteins in the free-swimming skin mucus, 113 unique proteins in the terrestrialized skin mucus, and 10 unique proteins in the terrestrialized gill mucus (Figure 3B). Analysis of the protein composition using the SRA-translated transcriptome resulted in 44 common proteins among all three samples, 190 unique proteins in the free-swimming skin mucus, 171 unique proteins in the terrestrialized skin mucus, and 11 unique proteins in the terrestrialized gill mucus (Figure 3C). A similar trend was observed when using the 454 translated transcriptome with 33 shared proteins among all three mucus samples, 130 proteins unique to the free-swimming skin mucus, 101 proteins unique to the terrestrialized skin mucus, and only 14 proteins unique to the terrestrialized gill mucus (Figure 3D). These results suggest that the composition of the gill mucus secretion produced during the induction phase of terrestrialization resembles both the free-swimming skin mucus and the terrestrialized skin mucus proteome. Specifically, ~72.9% of the terrestrialized gill mucus proteome was also present in the free-swimming skin mucus proteome and ~71.7% of the terrestrialized gill mucus proteome were found in the terrestrialized skin mucus. As a result, only ~16.4% of all proteins present in the terrestrialized gill mucus were unique to this sample.

**Figure 4:**
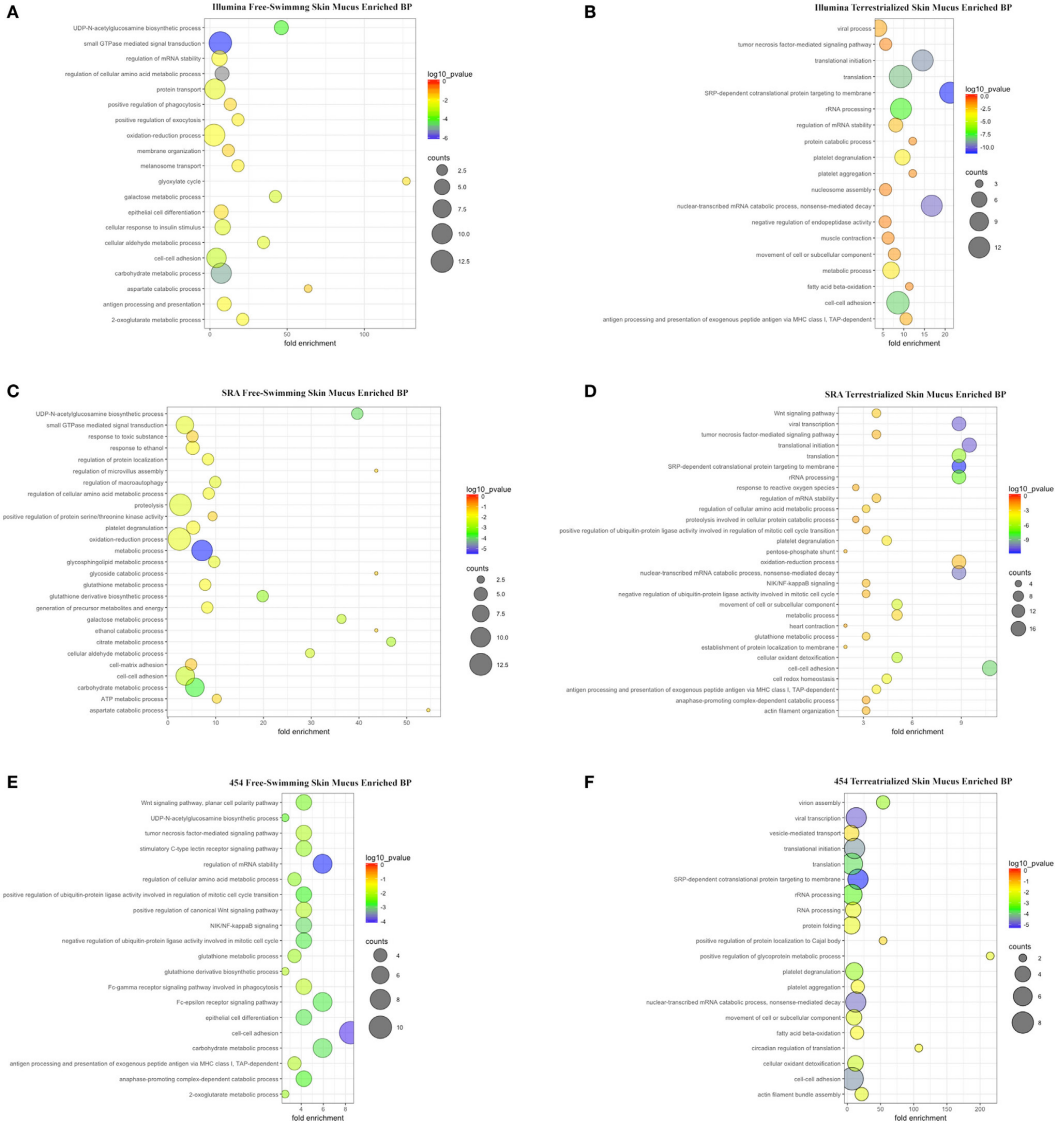
Scatter plots of enriched gene ontology biological process (BP) terms for **(A)** free-swimming skin mucus and **(B)** terrestrialized skin mucus using the Illumina database; for **(C)** free-swimming skin mucus and **(D)** terrestrialized skin mucus using the sequence read archive (SRA) database; and for **(E)** free-swimming skin mucus and **(F)** terrestrialized skin mucus using the 454 database. The fold enrichment indicates the ratio of the expressed gene number to the total gene number in a pathway. Only the top 20 BPs according to *p*-value are shown. The size and color of the points represent the gene number and the log10 *p*-value of each pathway, respectively.

Gene ontology analyses using the three data sets revealed that biological processes (BPs) were enriched in free-swimming and terrestrialized skin mucus samples, but no unique GO for the terrestrialized gill mucus (Table S4 in Supplementary Material; Figures 4A–F). Based on the Illumina analysis, the top five most significant BPs enriched in free-swimming skin mucus were “small GTPase mediated signal transduction,” “carbohydrate metabolic process,” “UDP-N-acetylglucosamine biosynthetic process,” “cell-cell adhesion,” and “galactose metabolic process.” In terrestrialized skin mucus, in turn, we observed an enrichment in BPs such as “SRP-dependent cotranslational protein targeting to membrane,” “viral transcription,” “nuclear-transcribed mRNA catabolic process, nonsense-mediated decay,” “translational initiation,” and “translation” (Figures 4A,B). Interestingly, all data sets also contained significant BPs related to immune function that were different in the free-swimming and terrestrialized skin mucus. For instance, free-swimming skin mucus had unique proteins involved in “positive regulation of phagocytosis” and “antigen processing and presentation,” whereas the terrestrialized skin mucus proteome included proteins with predicted functions in “platelet degranulation,” “antigen processing and presentation of exogenous peptide antigen *via* MHC class I, TAP-dependent,” “viral process,” “tumor necrosis factor-mediated signaling pathway,” “defense response to Gram-negative bacterium,” and “cellular response to hydrogen peroxide” (Table S4 in Supplementary Material). Overall, these data suggest that the immunological processes that govern the skin immune system in lungfish differ in free-swimming and terrestrialized phases. Additionally, non-immune BPs that were enriched in the control skin mucus included “epithelial cell differentiation” and “membrane organization,” while in terrestrialized skin mucus they included “translational initiation” and “platelet aggregation.” These results may indicate that cellular organization and maintenance functions are enriched in the skin of free-swimming animals, while cell survival is enriched during terrestrialization.

KEGG pathway analyses showed that, overall, in free-swimming lungfish skin mucus, enriched pathways are mostly related to metabolic pathways such as “Histidine metabolism,” “Amino sugar and nucleotide sugar metabolism,” “Galactose metabolism,” and “beta-Alanine metabolism.” The proteome of the terrestrialized lungfish skin mucus was predicted to be enriched in “Ribosome” and “Biosynthesis of antibiotics” pathways (Figure S1 in Supplementary Material). KEGG pathway analyses using the Illumina and SRA data sets (Figures S1A–D in Supplementary Material) were more similar to each other compared to the 454-derived KEGG pathway analysis (Figures S1E,F in Supplementary Material). These results support previous studies that demonstrated that terrestrialization has profound effects on lungfish skin metabolism with an overall decrease in metabolic activity.

### Changes in Lungfish Skin Immunoproteome in Response to Terrestrialization

**Figure 5:**
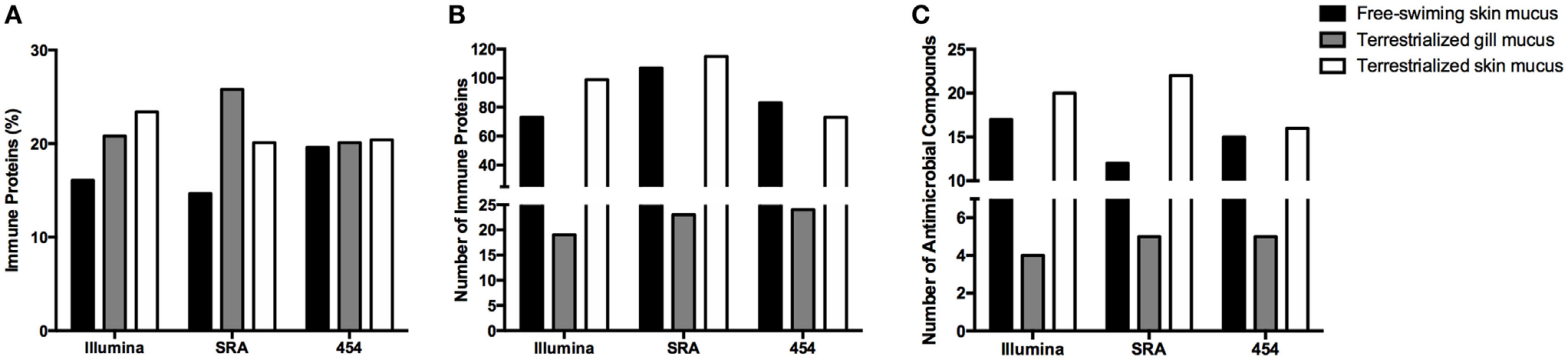
**(A)** Percentage of proteins and **(B)** total number of proteins with known immunological function in the three mucus samples obtained in this study using the Illumina transcriptome, the sequence read archive (SRA) transcriptome, or the 454 transcriptome databases. **(C)** Total number of proteins with known antimicrobial function in the skin identified in the three mucus samples using the Illumina transcriptome, SRA transcriptome, or the 454 transcriptome databases.

**Figure 6:**
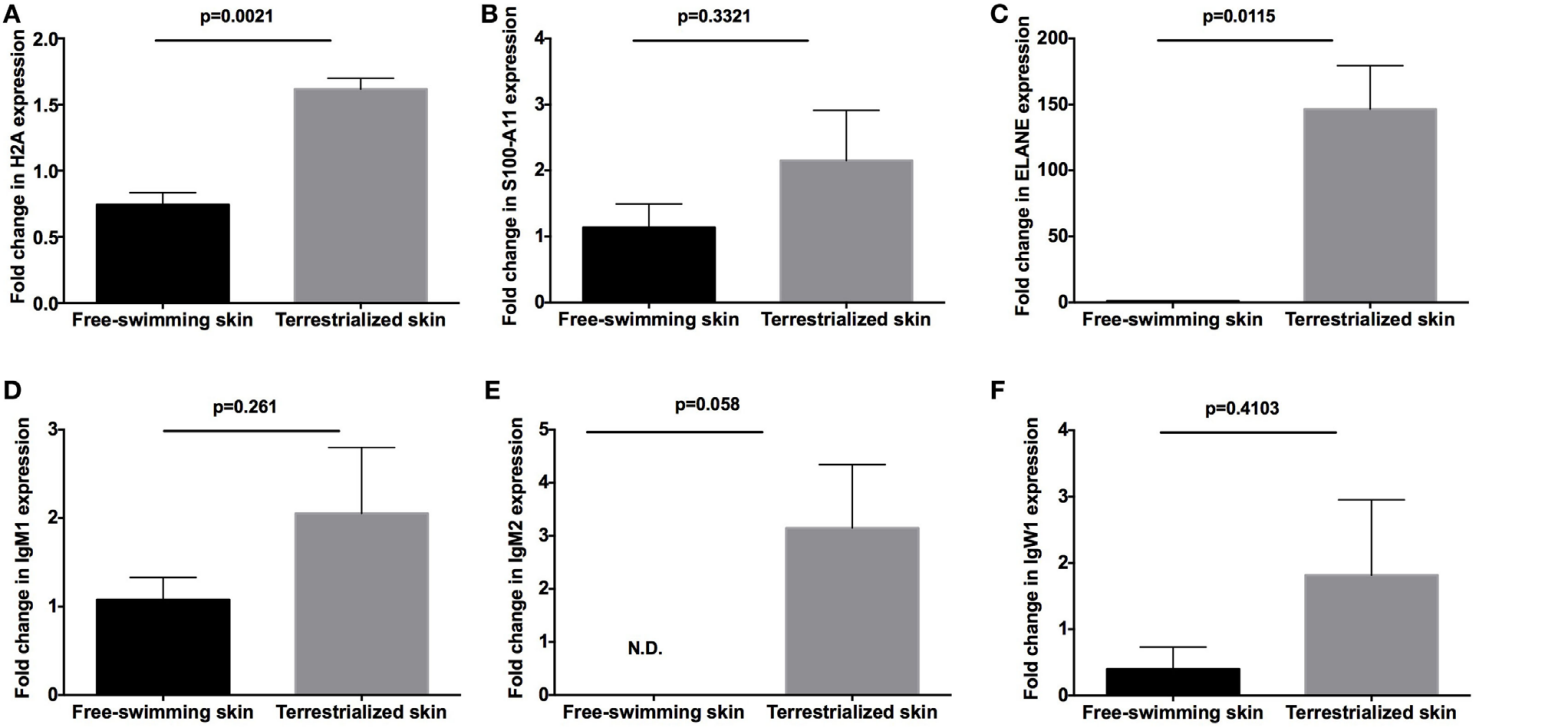
Quantitative real-time PCR analysis of the expression of **(A)** H2A, **(B)** S100-A11, **(C)** neutrophil elastase (ELANE), **(D)** IgM1, **(E)** IgM2, and **(F)** IgW1 in free-swimming and terrestrialized *Protopterus dolloi* skin tissue (N = 3). *p*-Values were calculated by unpaired *t*-test.

In order to examine the allocation of immune resources in lungfish skin in response to terrestrialization, we performed manual counts within our protein lists using previously reported proteins with immune function. We observed an increase in the percentage of immune-related proteins present in the terrestrialized gill and skin mucus compared to the free-swimming skin mucus when using lists generated by the Illumina and SRA data sets but not the 454 data set (Figure 5A). It is worth noting that the SRA-based analysis revealed a higher percentage of immune-related proteins in the gill mucus (~25%) compared to the Illumina and 454 data sets (20% in both). Similarly, when we counted the number of immune proteins that were present in each sample we found a trend toward greater number of immune proteins in the terrestrialized skin mucus compared to free-swimming skin mucus when using the Illumina and SRA data sets but not the 454 data set (Figure 5B). Among the immune-related proteins, we observed a higher number of proteins with known antimicrobial activity in the terrestrialized skin mucus compared to the free-swimming skin mucus in all three data sets (Figure 5C). The greater number of proteins with antimicrobial activity in the terrestrialized skin mucus sample was due to a greater abundance of histones and S100 proteins (Tables S1–S3 in Supplementary Material). In support, we observed increased levels of expression of H2A and S100A11 in terrestrialized compared to free-swimming skin by RT-qPCR (Figures 6A,B). Moreover, gene expression analysis of neutrophil elastase (ELANE) showed a significant increase (150-fold) in the expression of this gene in terrestrialized compared to free-swimming lungfish skin (Figure 6C). This result supports our histological observations as well as the proteomic results (Tables S1 and S2 in Supplementary Material). Combined, our results suggest that lungfish increase immune resource allocation and undergo inflammation in the skin early on during the aestivation process.

Apart from differences in antimicrobial compound abundance, we also observed changes in the Ig proteins present in each sample. Where no Igs were detected in the gill mucus sample, we detected IgW1 long form (IgW1L) and IgM1 in the free-swimming skin mucus sample and IgW1L, IgM1, and IgM2 in the terrestrialized skin mucus sample using the Illumina and 454 but not the SRA data sets (Tables S1–S3 in Supplementary Material). In support, RT-qPCR analysis showed no significant changes in IgW1 or IgM1 expression as a result of terrestrialization but a significant increase in IgM2 expression (~3-fold) was observed in terrestrialized compared to free-swimming skin (Figures 6D–F). Both sigma and lambda-like light chains were found in both mucus samples (Tables S1 and S2 in Supplementary Material). These results indicate a switch in Ig expression and secretion in the lungfish skin during the induction phase of aestivation.

## Discussion

The skin of all animals provides a first line of defense against pathogen invasion. Apart from being a physical barrier, the skin has its own unique suite of immune cells and molecules that constitute the skin-associated lymphoid tissue (39–40). As the major interface between the environment and the host, the skin is subject to several external stressors, and these stressors shifted dramatically during the vertebrate transition from water to land. Hence, we took advantage of the ability to terrestrialize lungfish in the laboratory setting as a model to study the water-to-land transition. We hypothesized that terrestrialization results in changes in the skin proteome composition and specifically, in an increased investment in production of immune molecules that will help prevent pathogen invasion and land stressors.

Previous studies have shown changes in the skin proteome composition of teleost fish (aquatic vertebrates) in response to infection (41, 42), stress (43–45), wounding (46), or dietary administration of immunostimulants (44, 45, 47). However, most of these studies adopted a two-dimensional (2-D) gel electrophoresis approach where selected spots were then analyzed by peptide fragment fingerprinting and LC-MS/MS. 2-D gel electrophoresis presents a number of drawbacks such as low reproducibility, the need for large sample sizes, and the difficulty to separate proteins with low abundance as well as very hydrophobic proteins (48). Thus, our results constitute a unique and unbiased report of all proteins present in the skin proteome of lungfish. We used three different RNA-seq databases from two different lungfish species (*P. dolloi* and *P. annectens*) generated in three different platforms (named Illumina, SRA, and 454) in our analyses. As expected, we obtained different results depending on the database and tissue origin. We found greater number of proteins in every sample when the SRA database was used, an expected result given the coverage of protein-coding sequences in each data base (636 unique sequences for the lungfish SRA database but only 494 for the Illumina database and 435 for the 454 database according to Swissprot).

As previously reported, we observed dramatic remodeling of the lungfish skin histological organization upon terrestrialization. This tissue remodeling involves multiple processes such as flattening of the epithelial cells, decreases in the overall epidermal thickness, and loss of goblet cells. In support, our proteomic study showed enriched BPs and KEGG pathways present in the skin mucus before and during terrestrialization highlighting the ability of this vertebrate to respond to environmental stimuli and reshape the cellular and molecular composition of the skin. One potential caveat to our study is that increased amounts of cell debris may be present in the skin mucus samples of terrestrialized animals than in free-swimming lungfish, affecting the overall proteomic composition of the two samples. In any case, tissue remodeling and changes in the external microbial environment likely occur concomitantly in our model and teasing apart the immunoproteome changes that respond to one or the other stimulus is a challenging question.

Transition from water to land also imposes drastic changes in the microbial composition of the external environment. Thus, we observed that lungfish secrete unique suites of innate and adaptive immune molecules into the skin mucus as a response to air exposure. With respect to innate immune molecules, we identified greater number of proteins with known antimicrobial functions in the skin, particularly histones (HIST1H1D and HIST1H3C) and S100 proteins (S100A, S100P, and S100A6). In this study, we confirmed changes at the protein level with gene expression data for six selected genes. Overall, gene expression data supported the findings of the proteomics approach, but it is worth noting that we did not include biological replicates in the proteomics study. Previous work has shown a lack of correlation between proteomics and mRNA transcript levels (49) and therefore future studies should expand our current data sets to multiple biological replicates. GO analyses of the unique proteins present in the terrestrialized skin mucus revealed enrichment in BPs such as defense response to Gram-negative bacterium, antigen processing, and presentation of exogenous peptide antigen *via* MHC class I, TAP-dependent, tumor necrosis factor-mediated signaling pathway, and cellular response to hydrogen peroxide. Thus, these data suggest a requirement for the lungfish skin to increase antimicrobial defenses during the process of terrestrialization. Future studies should address the function of antimicrobial compounds triggered by air exposure.

*Protopterus* sp. express four different immunoglobulin heavy (IgH) chain classes: IgW, IgM, IgN, and IgQ. Additionally, there is extensive intraclass IgH diversification in lungfish, with IgM including three IgM genes (IgM1, IgM2, and IgM3), IgW including two genes (IgW1 and IgW2) as well as short and long forms, and IgN including also three genes (IgN1, IgN2, and IgN3) (50). So far, the immunological functions of different IgM subclasses have not been investigated. This study identified the presence of Igs in the skin mucus proteome of both freshwater and terrestrialized lungfish. Specifically, the freshwater lungfish skin proteome contained IgW1L as well as IgM1 secretory form. Secreted IgW(D) antibodies have not been previously characterized in sarcopterygian fish, but it is known that teleost secrete IgD into their mucosal secretions (51). Our findings support that secretion of IgW into the skin mucus occurs in sarcopterygian fish and that IgW1L may have specialized mucosal immune functions compared to other IgW forms in lungfish. In the terrestrialized skin mucus proteome, apart from IgW1L, we observed both IgM1 and IgM2 expression, suggesting the IgM1 expression occurs constitutively in the skin of lungfish but IgM2 expression is switched on in response to external stressors. We were not able to detect any Ig in the terrestrialized gill mucus sample, but this finding may be a result of the lower protein amounts in this sample compared to the skin mucus samples. Further studies should address the specific immunological function of IgM2 at lungfish mucosal surfaces.

## Conclusion

This study provides the first characterization of the skin mucus proteome of a sarcopterygian fish, the African lungfish. We report important shifts in both innate and adaptive immune molecules in the skin mucus of lungfish in response to terrestrialization. Our results suggest that the transition from water to land in vertebrates imposed a need for increased investment in immune function in the cutaneous mucosal secretions.

## Data Availability

Proteomic data sets were submitted to ProteomeXchange (http://www.proteomexchange.org/) *via* the PRIDE database, accession: PXD008981 and PXD008982.SRA used in this study can be found at NCBI (accession numbers SRR2027914, SRR2027978, SRR2027979, SRR2028000, SRR2028017, SRR2028020, SRR2028021, SRR2027980, SRR6291329, and SRR6291330). 454 pyrosequencing reads were deposited at NCBI (accession number SRP141470). Output tables from Scaffold containing all peptide table reports are given in Table S5 in Supplementary Material.

## Ethics Statement

All animal studies were reviewed and approved by the Office of Animal Care Compliance at the University of New Mexico (protocol number 11-100744-MCC).

## Author Contributions

IS conceived the experiments. RH was responsible for the lungfish aestivation and performed histology, protein extraction, and transcriptomic data analysis. EC performed the RT-qPCR analysis. RH and IS wrote the manuscript and created the figures. All authors reviewed and revised the manuscript and approved the final manuscript prior to being submitted.

## Conflict of Interest Statement

The authors declare that the research was conducted in the absence of any commercial or financial relationships that could be construed as a potential conflict of interest.
