# Supplementary figures and images for: Effects of Experimental Terrestrialization on the Skin Mucus Proteome of African Lungfish (Protopterus dolloi)
Source: Front Immunol. 2018 Jun 4;9:1259. doi: 10.3389/fimmu.2018.01259 (PMC5994560; doi:10.3389/fimmu.2018.01259)

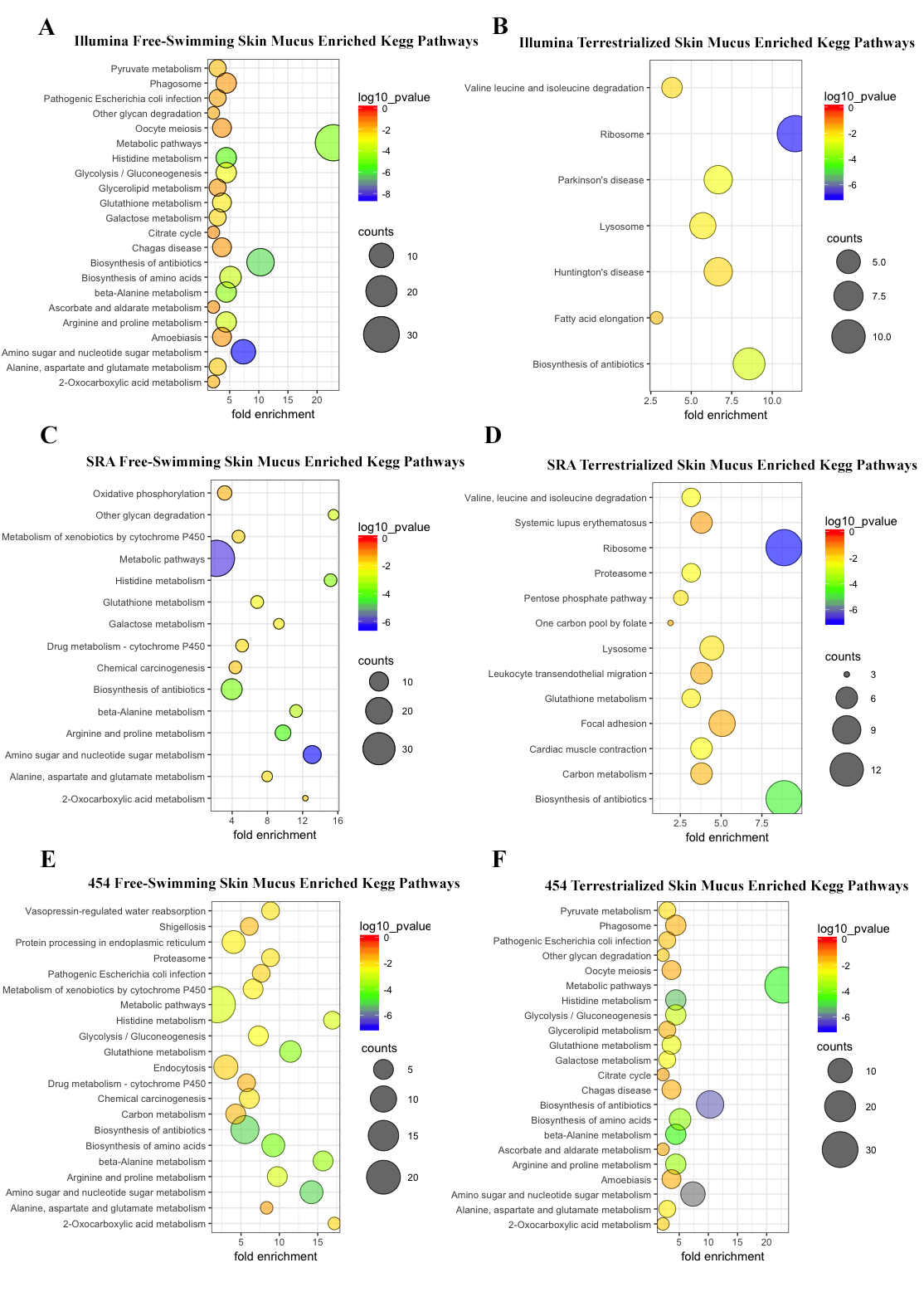

Supplement: Supplementary file 6 [file Image_1.TIFF]
